# Cardiovascular mortality trends in Switzerland 1995–2018

**DOI:** 10.1093/eurpub/ckac164

**Published:** 2022-11-09

**Authors:** Lisa Sorrentino, Arnaud Chiolero, Cristian Carmeli

**Affiliations:** Population Health Laboratory (#PopHealthLab), University of Fribourg, Fribourg, Switzerland; Population Health Laboratory (#PopHealthLab), University of Fribourg, Fribourg, Switzerland; School of Population and Global Health, McGill University, Montreal, Canada; Population Health Laboratory (#PopHealthLab), University of Fribourg, Fribourg, Switzerland

## Abstract

Mortality rates due to coronary heart disease (CHD) and stroke have declined in the last century in high-income countries, including Switzerland. However, these rates have plateaued in several countries. We assessed CHD and stroke mortality trends (1995–2018) in Switzerland. We estimated annual rate changes via JoinPoint regression. Rates decreased steadily in most sex and age groups; however, in those aged 60–74, stroke rates plateaued after 2012 among men and CHD rates plateaued after 2015 among women. Cardiovascular mortality continues to decrease in most of the Swiss population. Prevention efforts should be maintained, especially in individuals aged 60–74.

## Introduction

While cardiovascular diseases (CVD), including coronary heart disease (CHD) and stroke, are among the leading causes of death worldwide, related mortality rates have greatly declined in Europe and North America in the last century.^1^ These decreases can be explained by the large-scale use of evidence-based CVD treatments, improved health care management of hypertension and hypercholesterolemia, as well as the mitigation of CVD risk factors at a population level through public health measures.^1^

Recent studies conducted in several high-income countries have reported that the decrease in CVD mortality rates might have plateaued after 2000, possibly due to an increase in certain risk factors such as diabetes and obesity. Specifically, a study of CHD mortality rates in the USA observed a plateau from 2000 to 2002 in both men and women aged 35–54.^2^ A study of 26 European countries^3^ reported a plateau in CHD mortality rates in young adults (<45 years [years]) between 2000 and 2009 across eight countries. Further, a study of mortality before the age of 75 years in 23 high-income countries observed an increase in CVD mortality rates from 2014 for both sexes in the USA, and for women in Canada.^4^

In Switzerland, one previous study has shown that CVD mortality rates in the age group 35–74 years were consistently decreasing between 2000 and 2015, for both sexes.^4^ In the current study, we aimed to update and refine these trend analyses using data collected until 2018, across more specific age groups and focusing on CHD and stroke mortality. Further, we applied a data-driven approach, without imposing *a priori* a cut-off year for assessing potential variations in the annual change of rates.

## Methods

We conducted a population-based analysis of all CHD and stroke deaths in Switzerland between the years 1995 and 2018. Data about specific causes of death, age at death, sex and population size which was aggregated per year were extracted from the mortality statistics database of the Swiss Federal Statistical Office.

Underlying causes of death were recorded via the International Classification of Diseases (ICD10) coding system. We selected the ICD10 codes of I20–I25 and I60–I69, corresponding to CHD and stroke, respectively. Most of these diseases share the same etiology and risk factors (e.g. hypertension, diabetes, hypercholesterolemia, obesity, tobacco, unhealthy diet and low physical activity),^5^ and are among the major causes of mortality in Switzerland. We computed CHD, stroke and their sum (CHD+Stroke) age-standardized mortality rates stratified by sex and by specific age groups encompassing all ages (All), younger than 45 years (0–44), from 45 years to 59 years (45–59), from 60 years to 74 years (60–74), and older than 75 years (75+). Findings examining age groups of 10 yr intervals provided similar results. We implemented in R software (version 4.4.0) the direct standardization method with the 1976 European standard population as a reference.

To assess dynamics in mortality trends, we estimated the potential variability of annual percentage change (APC) in mortality rates using segmented regression (JoinPoint Regression Program, version 4.8.0.1).^6^ The JoinPoint software divided the 1995–2018 period into a maximum of four segments, chunks of calendar years and fit a linear model to the log-transformed mortality rates within each of these segments, whereby the slope parameter corresponded to the APC value in mortality rates.^7^ The optimal number of segments was selected by testing the null model with no joinpoints against a model with one or more joinpoints via 4500 Monte Carlo permutations at a significance level of 5%. Models with either no joinpoints, corresponding to a constant APC across the period 1995–2018, or one joinpoint, corresponding to two APC values, were finally selected. Uncertainty in the estimated APC values was calculated via bootstrap resampling (1000 replications) and reported with 95% confidence intervals (CI).

## Results

**Figure 1:**
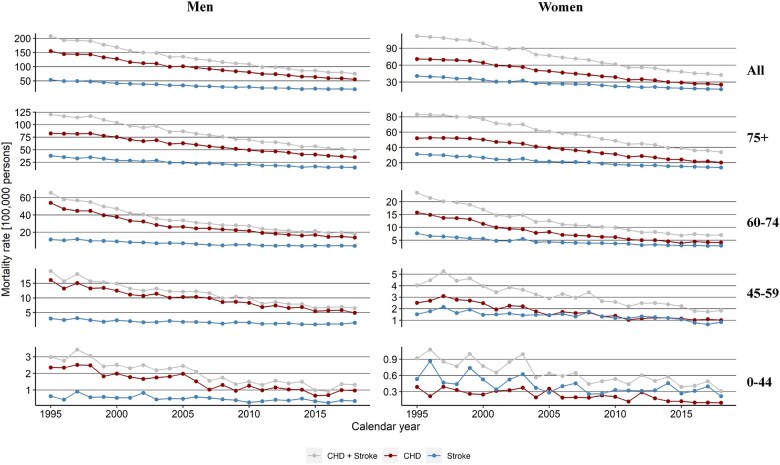
Trends of coronary heart disease (CHD) and stroke mortality rates in Switzerland by sex and age, 1995–2018. Age groups (from top to bottom: All, 75+, 60–74, 45–59, 0–44) are in rows, while sex is in columns (men on the left and women on the right). The age-standardized rates were estimated yearly. CHD+Stroke mortality rates are the sum of CHD and stroke mortality rates

Age-standardized mortality rates for CHD and stroke decreased between 1995 and 2018 in all age groups and both sexes (figure 1). When considering mortality of both CHD and stroke together for all ages, among men the rates decreased from 208 per 100 000 persons in 1995–75 per 100 000 persons in 2018, while among women they declined from 112 to 43, corresponding to a relative decrease of 64% in men and 61% in women. Over this period, the contribution of both CHD and stroke to the total mortality burden decreased substantially, accounting for 25% of total deaths for men and 26% for women in 1995, and 16% for men and 14% for women in 2018, respectively.

The JoinPoint analysis showed APC was constant or did not attenuate between 1995 and 2018 in most combinations of sex and age groups. For example, at all ages with mortality cause considered together (Supplementary table S1), the APC was −4.4 (95% CI −4.5 to −4.3) among men, and of similar size among women. However, more complex trends were observed in the age stratum 60–74 years. Specifically, for CHD the APC attenuated from −7.1 (95% CI −7.9 to −6.2) in the period 1995–2004 to −4.7 (95% CI −5.1 to −4.2) in the period 2005–2018 among men, while the APC attenuated from −6.5 (95% CI −6.8 to −6.1) in the period 1995–2015 −0.4 (95% CI −6.1 to 7.3) in the period 2016–18 among women (Supplementary table S2). For stroke, the APC attenuated from −5.6 (95% CI −6.3 to −5.0) in the period 1995–2012 to −0.8 (95% CI −4.0 to 2.5) in the period 2013–18 only among men (Supplementary table S3).

## Discussion

Mortality rates due to CHD and stroke have decreased substantially in Switzerland from 1995 to 2018, across the young and the old, and for men and women. However, in the age range 60–74 years, stroke mortality rates plateaued after 2012 among men and the reduction in CHD mortality rates plateaued after 2015 among women.

The steady decrease in CHD and stroke mortality rates across most sex and age groups is in line with a previous analysis over the period 2000–15.^4^ We did not observe a slowing or a plateauing in trends of CHD and stroke mortality rates in the young as reported in previous studies from other countries, as the USA and Italy.^2–4^ The decline in CHD and stroke mortality rates during the 1995–2018 period was much bigger than the decrease in cancer mortality rates during the same period in Switzerland.^8^

A potential limitation of our study may arise from time-varying misclassification in the cause of death due to garbage codes, namely ICD10 codes with ill-defined causes of death like essential hypertension or cardiac arrest. The presence of ICD10 garbage codes lowers the proportion of deaths attributed to CHD.^9^ An underestimation for women is also possible as CHD is less diagnosed due to a different presentation and the risk factors are underdiagnosed.^10^ Finally, the plateau seen in CHD mortality for women since 2015 covered a limited number of calendar years and may be due to the overfitting of the JoinPoint regression modeling. Consequently, this finding should be replicated in subsequent studies.

Our findings contribute to a call for public health actions to maintain and strengthen the prevention of cardiovascular mortality in the Swiss population, especially in individuals aged 60–74, and for continued surveillance of cardiovascular mortality in Switzerland.

## Supplementary data

Supplementary data are available at *EURPUB* online.

## Conflict of interest

None declared.

## Supplementary Material

ckac164_Supplementary_Data

## Data Availability

The data underlying this article were provided by the Swiss Federal Statistical Office under licence/by permission. Data will be shared on request to the corresponding author with the permission of the Swiss Federal Statistical Office. Cardiovascular mortality rates have stopped declining in many high-income countries at the turn of the millennium. We assessed coronary heart diseases (CHD) and stroke mortality rates trends between 1995 and 2018 in Switzerland. Rates decreased steadily in most sex and age groups, while among the 60–74 years old, stroke rates plateaued after 2012 among men and CHD rates plateaued after 2015 among women. Cardiovascular mortality continues to decrease in most of the Swiss population and prevention efforts should be maintained, especially in individuals older than 60 years.
