# Supplementary material for: Cardiovascular mortality trends in Switzerland 1995–2018
Source: Eur J Public Health. 2022 Nov 9;32(6):891–3. doi: 10.1093/eurpub/ckac164 (PMC9713456; doi:10.1093/eurpub/ckac164)
Supplement: ckac164_Supplementary_Data [file ckac164_supplementary_data.pdf]

Supplementary material

Supplementary Table 1. Annual Percentage Change (APC) of coronary heart diseases + stroke mortality rates. APC are shown for all age groups (rows) and sex (columns), with 95% confidence intervals (CI).

**Age range**	**Men**<br>**APC (95% CI)**	**Women**<br>**APC (95% CI)**
**All ages**	Period: 1995–2018<br>-4.4 (-4.5, -4.3)	Period: 1995–1999<br>-2.2 (-4.2, -0.1)<br>Period: 2000–2018<br>-4.6 (-4.8, -4.4)
**75+**	Period: 1995–1998<br>-1.5 (-4.7, 1.9)<br>Period: 1999–2018<br>-4.1 (-4.3, -4.0)	Period: 1995–1999<br>-0.9 (-2.9, 1.2)<br>Period: 2000–2018<br>-4.5 (-4.7, -4.3)
**60–74**	Period 1995–2004<br>-6.9 (-7.8, -6.0)<br>Period 2005–2018<br>-4.5 (-5.0, -4.1)	Period 1995–2015<br>-5.7 (-6.0, -5.3)<br>Period 2016–2018<br>0.3 (-6.1, 7.1)
**45–59**	Period 1995–2018<br>-4.5 (-4.9, -4.1)	Period 1995–2018<br>-4.1 (-4.7, -3.6)
**0–44**	Period 1995–2018<br>-4.6 (-5.4, -3.8)	Period 1995–2018<br>-4.0 (-5.0, -3.0)

Supplementary Table 2. Annual Percentage Change (APC) of coronary heart diseases mortality rates. APC are shown for all age groups (rows) and sex (columns), together with 95% confidence intervals for coronary heart disease (ICD10 codes: I20–I25).

**Age range**	**Men**<br>**APC (95% CI)**	**Women**<br>**APC (95% CI)**
**All ages**	Period: 1995–2018<br>-4.4 (-4.6, -4.3)	Period: 1995–1999<br>-1.2 (-3.3, 1.0)<br>Period: 2000–2018<br>-5.1 (-5.3, -4.9)
**75+**	Period: 1995–1998<br>-0.2 (-3.2, 2.9)<br>Period: 1999–2018<br>-4.1 (-4.3, -4.0)	Period: 1995–2000<br>-0.6 (-2.1, 1.0)<br>Period: 2001–2018<br>-5.0 (-5.2, -4.8)
**60–74**	Period: 1995–2004<br>-7.1 (-7.9, -6.2)<br>Period: 2005–2018<br>-4.7 (-5.1, -4.2)	Period: 1995–2015<br>-6.5 (-6.8, -6.1)<br>Period: 2016–2018<br>0.4 (-6.1, 7.3)
**45–59**	Period: 1995–2018<br>-4.6 (-5.0, -4.2)	Period: 1995–2018<br>-4.9 (-5.5, -4.2)
**0–44**	Period: 1995–2018<br>-5.0 (-6.0, -4.1)	Period: 1995–2018<br>-5.2 (-6.8, -3.7)

Supplementary Table 3. Annual Percentage Change (APC) of stroke mortality rates. APC are shown for all age groups (rows) and sex (columns), together with 95% confidence intervals for stroke (ICD10 codes: I60–I69).

**Age range**	**Men**<br>**APC (95% CI)**	**Women**<br>**APC (95% CI)**
**All ages**	Period: 1995–2014<br>-4.5 (-4.7, -4.3)<br>Period: 2015–2018<br>-1.9 (-4.5, 0.8)	Period: 1995–2018<br>-3.7 (-3.9, -3.5)
**75+**	Period: 1995–2018<br>-4.2 (-4.4, -3.9)	Period: 1995–2018<br>-3.7 (-3.8, -3.5)
**60–74**	Period: 1995–2012<br>-5.6 (-6.3, -5.0)<br>Period: 2013–2018<br>-0.8 (-4.0, 2.5)	Period: 1995–2018<br>-4.0 (-4.4, -3.7)
**45–59**	Period: 1995–2018<br>-4.0 (-4.9, -3.1)	Period: 1995–2013<br>-2.0 (-3.1, -0.8)<br>Period: 2014–2018<br>-11.0 (-18.1, -3.3)
**0–44**	Period: 1995–2018<br>-3.3 (-4.6, -1.9)	Period: 1995–2018<br>-3.4 (-4.9, -1.8)
